# Temporal RAGE Over-Expression Disrupts Lung Development by Modulating Apoptotic Signaling

**DOI:** 10.3390/cimb46120867

**Published:** 2024-12-21

**Authors:** Derek M. Clarke, Madison N. Kirkham, Logan B. Beck, Carrleigh Campbell, Hayden Alcorn, Benjamin T. Bikman, Juan A. Arroyo, Paul R. Reynolds

**Affiliations:** Department of Cell Biology and Physiology, Brigham Young University, 3054 Life Sciences Building, Provo, UT 84602, USA

**Keywords:** RAGE, lung, embryo, transgenic, apoptosis

## Abstract

Receptors for advanced glycation end products (RAGE) are multiligand cell surface receptors found most abundantly in lung tissue. This study sought to evaluate the role of RAGE in lung development by using a transgenic (TG) mouse model that spatially and temporally controlled RAGE overexpression. Histological imaging revealed that RAGE upregulation from embryonic day (E) 15.5 to E18.5 led to a thickened alveolar parenchyma and reduced alveolar surface area, while RAGE overexpression from E0 to E18.5 caused a significant loss of tissue and decreased architecture. Mitochondrial dysfunction was a hallmark of RAGE-mediated disruption, with decreased levels of anti-apoptotic BCL-W and elevated pro-apoptotic BID, SMAC, and HTRA2, indicating compromised mitochondrial integrity and increased intrinsic apoptotic activity. Extrinsic apoptotic signaling was similarly dysregulated, as evidenced by the increased expression of TNFRSF21, Fas/FasL, and Trail R2 in E0-18.5 RAGE TG mice. Additionally, reductions in IGFBP-3 and IGFBP-4, coupled with elevated p53 and decreased p27 expression, highlighted disruptions in the cell survival and cycle regulatory pathways. Despite the compensatory upregulation of inhibitors of apoptosis proteins (cIAP-2, XIAP, and Survivin), tissue loss and structural damage persisted. These findings underscore RAGE’s role as a pivotal modulator of lung development. Specifically, the timing of RAGE upregulation significantly impacts lung development by influencing pathways that cause distinct histological phenotypes. This research may foreshadow how RAGE signaling plausibly contributes to developmental lung diseases.

## 1. Introduction

Lung development in mammals is a meticulously orchestrated process that progresses through distinct stages—pseudoglandular, canalicular, saccular, and alveolar—culminating in a functional respiratory system at birth. This progression involves precise cellular differentiation, extracellular matrix remodeling, and the formation of intricate airway and alveolar structures essential for effective gas exchange [1]. These stages involve complex interactions between epithelial and mesenchymal cells, the coordinated remodeling of the extracellular matrix, and finely tuned cell proliferation and apoptosis that together establish functional respiratory structures. A particularly critical aspect of this developmental program is the regulation of apoptosis, which ensures the removal of unnecessary or improperly positioned cells, thereby refining lung architecture [2].

The Receptor for Advanced Glycation End Products (RAGE) has emerged as a notable contributor in lung development. Known for its involvement in inflammatory and chronic disease pathways, RAGE is also highly expressed in the lung, particularly by alveolar epithelial cells, and its expression pattern suggests a role in lung formation [3]. Previous studies in our lab have demonstrated that RAGE signaling is critical during alveolarization, where it may influence cellular differentiation and apoptosis—key processes in the final stages of lung organogenesis [4]. RAGE’s role in development appears to extend beyond lung morphogenesis, with evidence suggesting that it functions as a multi-ligand receptor influencing cellular responses in various tissues, likely through pathways associated with NF-κB and MAPK signaling [5]. Notably, the overexpression of RAGE in transgenic mouse models has been associated with altered lung development, including changes in mitochondrial function and bioenergetics [6,7].

Apoptosis is a fundamental process in lung development that facilitates the removal of excess or misplaced cells to refine lung architecture. Specifically, apoptosis plays a pivotal role during alveolar formation, where it helps to shape the delicate balance of cellular populations needed for optimal gas exchange [8]. The inappropriate regulation of apoptosis can lead to developmental abnormalities or impaired lung function, highlighting its critical role in organogenesis [9]. Recent work has suggested a possible link between RAGE signaling and apoptotic pathways, as the ligation of RAGE has been shown to influence mitochondrial integrity and the activation of pro-apoptotic signaling molecules essential to the apoptotic process [4].

This study aimed to elucidate the role of RAGE in lung development by examining its impact on proteins implicated in mitochondrial stability and apoptotic pathways. By investigating transgenic mice that overexpress RAGE specifically in the lung, we sought to understand how RAGE influences mitochondrial integrity, apoptosis regulation, and overall lung morphogenesis. Our findings may provide insights into the molecular mechanisms underlying lung development and the potential implications of RAGE signaling in pulmonary diseases.

## 2. Methods

### 2.1. Animals

All mice had a C57Bl/6 background. Controllable RAGE overexpression was accomplished by mating two transgenic mouse lines via the doxycycline (dox)-inducible mechanism, as described previously [4,10]. Specifically, these RAGE TG mice employed the expression of a reverse tetracycline transactivator (rtTA) induced by the surfactant protein C promoter and the resulting lung-specific rtTA complexed with Dox to activate the RAGE gene downstream from TetOn elements [10]. PCR genotyping determined the existence of transgenes from tail biopsies. Two groups of eight time-mated pregnant mice were provided dox feed (625 mg/kg; Invigo Teklad, St. Louis, MO, USA) during a short-term treatment from embryonic day (E) 15.5 until E18.5 or throughout development via dox feeding from E0 to E18.5. Control mice treated with Dox were also included in the experimental design. Lungs from double-transgenic (TG) and littermate non-transgenic controls (from at least 4 different pups each) were procured at E18.5 and processed, embedded in paraffin, and then sectioned as already described [4,10]. Additional lungs were also procured from mice at E18.5 (from at least 4 different TG and controls in each group) and total homogenates were obtained. All mice were housed and used in accordance with a breeding and experimental protocol approved by the Institutional Animal Care and Use Committee at Brigham Young University (21-0202 and 21-0203), approved on 16 March 2021.

### 2.2. Histology

Lungs from the control and TG mice were processed and embedded in paraffin. Lungs were sectioned and prepared for staining using hematoxylin and eosin to visualize their basic morphology. Stained sections were imaged using the Olympus BX51 microscope (Waltham, MA, USA).

### 2.3. Apoptotic Signaling Analyses

The total protein in homogenized lungs was quantified using a BCA Protein Assay Kit (Thermo Fisher Scientific, Waltham, MA, USA). A mouse apoptosis antibody array C1 (RayBiotech, Norcross, GA, USA) was performed as previously described in our laboratory [11]. Briefly, 125 μg of total protein lysate per sample (control and TG for a total of n = 8 per group) was collected and divided to create two sample pools, each with a concentration of 500 μg/mL (protein samples from 4 animals were pooled for each blot; *n* = 4 animals per blot). Two sample pools per group were added to individual membranes and incubated overnight. Biotinylated antibodies were then added to each membrane, followed by a final incubation with a streptavidin-conjugated fluorescent label (Thermo Fisher Scientific, Waltham, MA, USA) to detect cytokine expression. Membranes were imaged using the Odyssey DLx Near-Infrared Fluorescence Imaging System (LI-COR, Lincoln, NE, USA). The results were quantitatively analyzed using Image J v1.54 (U.S. National Institutes of Health, Bethesda, MD, USA, accessed 13 October 2024).

### 2.4. Statistical Analysis

GraphPad Prism software (GraphPad version 10.1.0; Santa Clara, CA, USA) was used for statistical analyses. Mean values ± S.E. per animal group were assessed by one- and two-way analysis of variance (ANOVA), while considering normal variances and variables. Mann–Whitney tests were employed, and the results exhibit significant differences where *p* values < 0.05.

## 3. Results

### 3.1. RAGE Upregulation and Altered Lung Morphology

**Figure 1:**
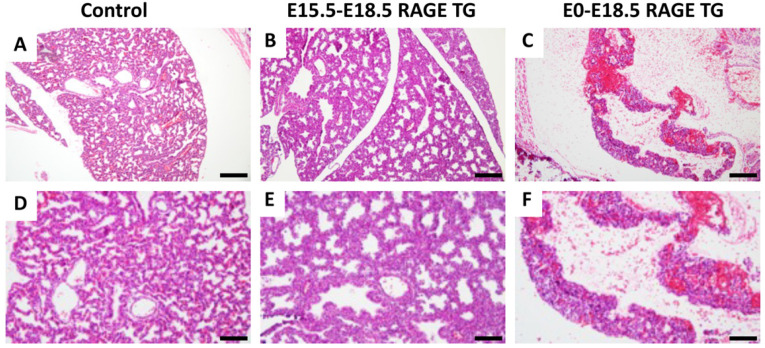
RAGE expression was upregulated in embryonic RAGE transgenic (TG) murine lung tissue, and hematoxylin and eosin stains were used to evaluate lung morphology. H&E-stained sections revealed the marked simplification of E0-E18.5 RAGE TG lungs (**C**,**F**) compared to controls (**A**,**D**). An intermediate phenotype was observed in E15.5-E18.5 TG lungs (**B**,**E**), in which tissue reduction resulted in fewer distal compartments surrounded by thickened alveolar walls. All images are representative. Original magnifications of 100× (**A**–**C**) and 200× (**D**–**F**) are shown, with scale bars representing 200 µm (**A**–**C**) or 100 µm (**D**–**F**).

Compared to the controls (Figure 1A,B), the H&E staining of RAGE TG lungs from E15.5-18.5 (Figure 1B,E) and E0-18.5 (Figure 1C,F) revealed significant developmental simplification. The lungs from TG mice that upregulated RAGE from E15.5-18.5 were primarily characterized by a thickened alveolar membrane and decreased alveolar surface area compared to the controls. Lungs with RAGE upregulation from E0-18.5 experienced significant lung hypoplasia compared to controls, with significantly less tissue and simplified respiratory structures (Figure 1E,F). Dox administration alone (control mice exposed to Dox) led to no histological differences in the control lungs when compared to non-treated mice [10].

### 3.2. Mitochondrial-Related Apoptotic Modulators

**Figure 2:**
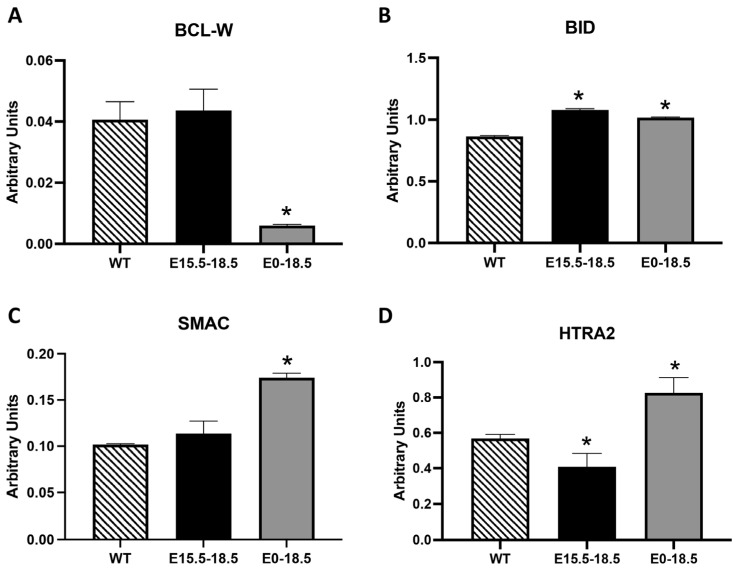
Differential expression of BCL-2 family members (BCL-W and BID) and mitochondrial proteins (SMAC and HTRA2) in RAGE TG mice. We discovered significantly less anti-apoptotic BCL-W (**A**) and significantly elevated levels of pro-apoptotic molecules including BID, SMAC, and HTRA2 (**B**–**D**) in E0-E18.5 RATE TG mouse lungs compared to the controls. While BLC-W and SMC were unchanged in E15.5-E18.5 RAGE TG mice, BID was markedly upregulated (**B**) and HTRA2 was significantly downregulated (**D**) compared to the controls. Significant differences when comparing the protein abundance in lung homogenates are noted as * *p* ≤ 0.05.

The BCL-2 family consists of both pro- and anti-apoptotic members that control mitochondrial membrane integrity and, consequently, the release of apoptotic factors like cytochrome c [12]. Furthermore, notable mitochondrial proteins facilitate apoptosis by neutralizing IAPs, which allows caspases to proceed through the cell death process [13]. We discovered significantly decreased BCL-W, a member of the BCL-2 family that helps maintain mitochondrial integrity and prevents the release of pro-apoptotic factors, in E0-18.5 RAGE TG mice compared to the controls (Figure 2A, *p* ≤ 0.05). In contrast, we detected the significant upregulation of pro-apoptotic BID in E15.5-18.5 and E0-18.5 RAGE TG mice (Figure 2B, *p* ≤ 0.05). Two pro-apoptotic molecules, namely SMAC, which inhibits IAPs, and HTRA,2, which degrades IAPs, were both upregulated in E0-18.5 RAGE TG mice (Figure 2C,D, *p* ≤ 0.05). Interestingly, HTRA2 was diminished in the acute E15.5-18.5 TG RAGE mice (Figure 2D, *p* ≤ 0.05).

### 3.3. Tumor Necrosis Factor (TNF) Family and Insulin-like Growth Factor Binding Proteins (IGFBPAs)

**Figure 3:**
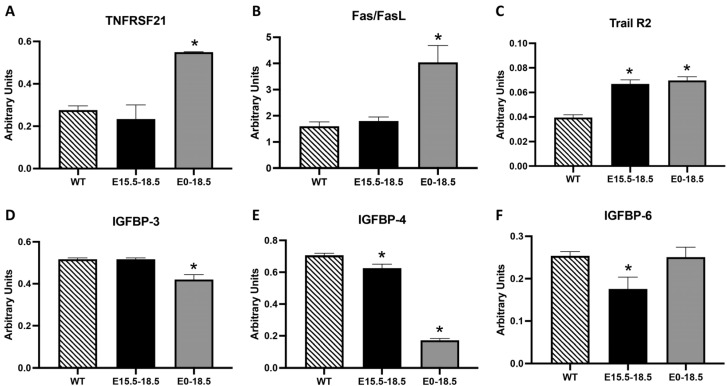
Differential expression of tumor necrosis factor superfamily and insulin-like growth factor-binding proteins (IGFBPs) in RAGE TG mice. Among the TNF family members, there was no significant difference in TNFRSF21 or Fas/FasL in E15.5-18.5 RAGE TG mice; however, Trail R2 was markedly increased in E15.5-18.5 RAGE TG mice compared to controls (**A**–**C**). Each of these pro-apoptotic factors (TNFRS21, Fas/FasL, and Train R2) were significantly increased in E0-18.5 RAGE TG mice compared to the controls (**A**–**C**). We discovered decreased IGFBP4 and IGFBP6 levels in E15.5-18.5 RAGE TG mice and significantly decreased IGFBP3 and IGFBP4 levels in E0-18.5 RAGE TG mice compared to the controls (**D**–**F**). Significant differences when comparing the protein abundance in lung homogenates are noted as * *p* ≤ 0.05.

The TNF family includes receptors that trigger the extrinsic apoptosis pathway in response to ligand binding [14]. We observed the significantly elevated expression of TNF family members that regulate pro-apoptotic effects, including TNFRSF21, Fas/FasL, and Trail R2 (Figure 3A–C, *p* ≤ 0.05), in E0-18.5 RAGE TG mice compared to the controls. Although TNFRSF21 and Fas/FasL were not elevated in E15.5-18.5 RAGE TG mice, Trail R2 was enhanced in lungs from E15.5-18.5 RAGE TG mice (Figure 3A–C, *p* ≤ 0.05). IGFBPs regulate IGF signaling, which is often associated with cell survival and the promotion of apoptosis, independent of IGF modulation [15]. We observed the decreased expression of IGFBP-3 and IGFBP-4 in E0-18.5 RAGE TG mice (Figure 3D,E, *p* ≤ 0.05) and the diminished expression of IGFBP-4 and IGFBP-5 in E15.5-18.5 RAGE TG mice (Figure 3E,F, *p* ≤ 0.05) compared to the controls.

### 3.4. Tumor Suppressor and Cell Cycle Regulators

**Figure 4:**
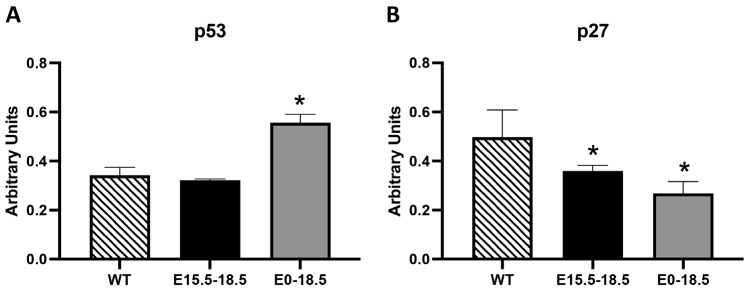
Differential expression of tumor suppressors and cell cycle regulators in RAGE TG mice. We discovered marked increases in the expression of p53, a key tumor suppressor protein that promotes apoptosis by activating pro-apoptotic genes, in E0-18.5 RAGE TG mice compared to the controls (**A**). We also observed a marked decrease in p27, a cyclin-dependent kinase inhibitor that regulates cell cycle progression, in both E15.5-18.5 and E0-18.5 RAGE TG mice compared to the controls (**B**). Significant differences when comparing the protein abundance in lung homogenates are noted as * *p* ≤ 0.05.

Tumor suppressors may initiate apoptosis as a response to stress, DNA damage, or oncogenic signals to prevent the proliferation of potentially cancerous cells [16]. We observed that p53 was significantly elevated in E0-18.5 RAGE TG mice (Figure 4A, *p* ≤ 0.05). This discovery supports the role of p53 as a key tumor suppressor protein that can promote apoptosis by activating pro-apoptotic genes and proteins. We also detected the differential expression of p27, a cyclin-dependent kinase inhibitor that regulates cell cycle progression. The observation that p27 was significantly decreased in both RAGE TG mice compared to the controls (Figure 4B, *p* ≤ 0.05) suggested cellular attempts to prevent or slow apoptotic progress.

### 3.5. Inhibitors of Apoptosis Proteins (IAPs) and Heat Shock Proteins (HSPs)

**Figure 5:**
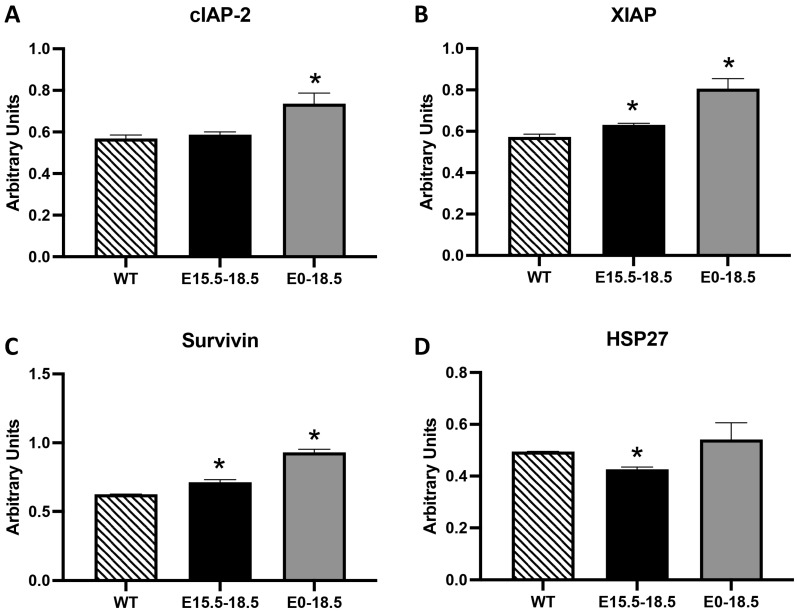
Differential expression of inhibitors of apoptosis proteins (IAPs) and heat shock proteins (HSPs) in RAGE TG mice. We detected elevated levels of XIAP and Survivin in both E15.5-18.5 and E0-18.5 RAGE TG mice compared to the controls (**B**,**C**), suggesting mechanisms that possibly attempt to prevent excessive cellular apoptosis. We similarly detected elevated levels of cIAP-2 in E0-18.5 RAGE TG mice compared to the controls (**A**). While HSP27 was unchanged in E0-18.5 RAGE TG mice, this molecular chaperone that protects cells from apoptosis was decreased in E15.5-18.5 RAGE TG mice compared to the controls (**D**). Significant differences when comparing the protein abundance in lung homogenates are noted as * *p* ≤ 0.05.

IAPs act as suppressors of apoptosis by directly inhibiting caspases, often ensuring cell survival under stress or damage conditions [17]. Heat shock proteins, particularly HSP27, act as anti-apoptotic agents by stabilizing mitochondrial membranes and other cellular structures [18]. We observed the highest expression of IAPs, including cIAP-2, XIAP, and Survivin (Figure 5A–C, *p* ≤ 0.05), in the E0-18.5 RAGE TG mice, which represented a period after significant tissue loss had already occurred (Figure 1E,F, *p* ≤ 0.05). Such attempts to stave off excessive RAGE-mediated apoptosis was also apparent in the elevated XIAP and Survivin levels observed in the E15.5-18.5 RAGE TG mice (Figure 5B,C, *p* ≤ 0.05). HSP27, which acts as a molecular chaperone that protects cells from apoptosis by inhibiting cytochrome c, was also decreased in E15.5-18.5 RAGE TG mice but unchanged in E0-18.5 TG mice (Figure 5D, *p* ≤ 0.05).

## 4. Discussion

The current study was focused on a gain-of-function model, aiming to understand the role of temporal RAGE overexpression throughout lung development. Accordingly, the findings presented here provide a comprehensive view of how RAGE overexpression influences lung morphogenesis. RAGE TG mice exhibited significant lung simplification, including thickened alveolar membranes and a reduced surface area, as shown in Figure 1. This phenotype was most pronounced in the E0-18.5 RAGE TG mice, where hypoplasia and structural disorganization were readily observed. These findings align with prior research indicating that aberrant RAGE signaling disrupts alveolar septation and differentiation, leading to morphogenetic defects [19]. The simplified architecture likely results from dysregulated cell turnover, including altered apoptosis and proliferation. Alveolar thickening and reduced surface area are consistent with diminished cellularity, which may impair gas exchange efficiency. These structural changes highlight the necessity for the precise temporal regulation of RAGE expression during development and suggests that early-life disruptions have more profound consequences than transient overexpression later in development.

Our data reveal significant alterations in mitochondrial-associated apoptotic modulators (Figure 2), implicating RAGE in the intrinsic pathway of apoptosis. The reduction in BCL-W, a BCL-2 family member critical for maintaining mitochondrial membrane integrity and preventing apoptosis, in E0-18.5 RAGE TG mice supports the hypothesis that RAGE disrupts mitochondrial homeostasis. This reduction likely contributes to increased mitochondrial membrane permeability and the release of pro-apoptotic factors such as cytochrome c, as previously described [12]. Conversely, the upregulation of BID, a pro-apoptotic BH3-only protein, further indicates that RAGE promotes mitochondrial dysfunction. BID activation is known to amplify apoptosis by facilitating mitochondrial outer membrane permeabilization [20]. The significant upregulation of SMAC and HTRA2, both of which antagonize IAPs, reinforces the role of RAGE in driving apoptosis by releasing mitochondrial proteins that enhance caspase activity [13].

The time-dependent differences in HTRA2 expression, elevated in E0-18.5 but reduced in E15.5-18.5 RAGE TG mice, suggests that RAGE exerts stage-specific effects on mitochondrial regulation. These findings align with studies highlighting the role of RAGE in modulating mitochondrial function and energy metabolism during development [7,21]. RAGE overexpression also significantly impacted extrinsic apoptotic pathways, as evidenced by the upregulation of TNF family members (Figure 3). The elevated levels of TNFRSF21, Fas/FasL, and Trail R2 in E0-18.5 RAGE TG mice indicate enhanced sensitivity to apoptotic signaling mediated by these death receptors. Fas/FasL interactions, for example, play a crucial role in immune-mediated apoptosis, while Trail R2 is associated with developmental apoptosis in epithelial tissues [14]. The selective upregulation of Trail R2 in E15.5-18.5 RAGE TG mice suggests that RAGE can amplify extrinsic apoptosis even during short-term overexpression.

Additionally, RAGE modulated the expression of IGFBPs (Figure 3). Reduced levels of IGFBP-3 and IGFBP-4 in E0-18.5 RAGE TG mice and IGFBP-4 and IGFBP-5 in E15.5-18.5 RAGE TG mice likely impair the pro-survival signaling typically associated with IGF modulation [15]. IGFBP-3, in particular, has been shown to induce apoptosis independently of IGF, suggesting that its downregulation may tip the balance toward cell survival under RAGE-mediated stress [22]. Such a conclusion may be supported by cellular attempts to prevent excessive apoptosis in an organ already numerically compromised in terms of cellularity.

In Figure 4, elevated p53 expression in E0-18.5 RAGE TG mice underscores its role as a tumor suppressor and stress response regulator. Increased p53 likely reflects attempts to limit the RAGE-driven proliferation of damaged cells, consistent with its activation in response to DNA damage and cellular stress [16]. Conversely, decreased p27 levels in both TG models suggest a failure to adequately enforce cell cycle arrest, potentially exacerbating apoptotic susceptibility. Furthermore, the observed increases in IAPs (cIAP-2, XIAP, Survivin; Figure 5) in E0-18.5 RAGE TG mice indicate a cellular attempt to counteract excessive apoptosis. However, the timing of these elevations suggests they are insufficient to prevent tissue loss, as cellular loss and structural damage have already occurred. Similarly, the reduction in HSP27 in E15.5-18.5 RAGE TG mice may further exacerbate apoptotic susceptibility by destabilizing mitochondrial membranes [18].

This study revealed that RAGE overexpression disrupts lung development through a combination of altered morphology, mitochondrial dysfunction, and heightened extrinsic apoptotic signaling. The temporal and molecular specificity of these effects underscores the critical role of RAGE in balancing apoptotic and survival pathways during morphogenesis. These findings accordingly provide valuable insights into the molecular mechanisms underlying RAGE-mediated developmental abnormalities and have potential implications for understanding its role in lung disease. Importantly, we assessed the factors described in the current study at E18.5, the date of necropsy. Therefore, such a specific snapshot at the end of the fetal period may be perceived as a limitation of this study. Future research into these factors at additional time points from E15 through E18 may provide an increased understanding of how apoptosis is modulated during development in response to RAGE expression. One such endeavor would show the possibility that RAGE-mediated inflammation is a key driver of tissue loss. Of particular interest in future research are the apoptosis-related effects of the nuclear signaling pathways JNK, AP-1, and Stat signaling, which are altered by RAGE in inflammatory pathways [23]. These pro-inflammatory intracellular signaling intermediates in the MAPK/AP-1 family perpetuate RAGE-induced genetic outcomes that lead to tissue loss [24]. A more exhaustive contextualization of these and other pathways may prove enlightening when characterizing perinatal lung anomalies such as bronchopulmonary dysplasia (BPD), acute respiratory distress syndrome (ARDS), and neonatal asthmatic phenotypes [25]. Such subsequent research has the potential for clinical significance due to enhancements in our understanding of the potential lung remodeling mechanisms encountered in BPD and ARDS specifically.

## Data Availability

All data are presented within the article. Data and other materials are available from the corresponding author upon reasonable request.
